# Barriers to initiating and maintaining participation in parkrun

**DOI:** 10.1186/s12889-022-12546-w

**Published:** 2022-01-13

**Authors:** L. J. Reece, K. Owen, M. Graney, C. Jackson, M. Shields, G. Turner, C. Wellington

**Affiliations:** 1 SPRINTER Research Group, Prevention Research Collaboration, School of Public health, Charles Perkins Centre, University of Sydney, Sydney, NSW Australia; 2 parkrun Global, London, UK

**Keywords:** Physical activity, Health, Community, Health inequalities

## Abstract

Interventions that increase population physical activity are required to promote health and wellbeing. parkrun delivers community-based, 5 km events worldwide yet 43% who register never participate in a parkrun event. This research had two objectives; i) explore the demographics of people who register for parkrun in United Kingdom, Australia, Ireland, and don’t initiate or maintain participation ii) understand the barriers to participating in parkrun amongst these people. Mandatory data at parkrun registration provided demographic characteristics of parkrun registrants. A bespoke online survey distributed across the three countries captured the reasons for not participating or only participating once. Of 680,255 parkrun registrants between 2017 and 19, 293,542 (43%) did not participate in any parkrun events and 147,148 (22%) only participated in one parkrun event. Females, 16–34 years and physically inactive were more likely to not participate or not return to parkrun. Inconvenient start time was the most frequently reported barrier to participating, with females more likely than males to report the psychological barrier of feeling too unfit to participate. Co-creating strategies with and for people living with a chronic disease, women, young adults, and physically inactive people, could increase physical activity participation within parkrun.

**Supplementary Information:**

The online version contains supplementary material available at 10.1186/s12889-022-12546-w.

## Background

The benefits of being physically active on health and wellbeing have been well researched and documented over the last five decades [1, 2]. To achieve health and wellbeing benefits, the World Health Organization (WHO) provide evidence-based public health recommendations for children, adolescents, adults and older adults on the recommended frequency, intensity and duration of physical activity [3]. Global guidelines like these are an essential component of a comprehensive governance and policy framework for public health action and provide a clear consistent global measurement framework of progress [3]. People who achieve physical activity guidelines are more likely to feel happier and healthier [4]. Despite this, more than 1.4 billion adults worldwide do not achieve the recommended levels of physical activity and inequities in participation are well documented [4].People from disadvantaged areas (low socio-economic status) are more likely to be physically inactive and are therefore at an elevated risk of developing chronic diseases [5], exacerbating health inequalities. The scale of this physical inactivity pandemic is clear, resulting in 5 million global deaths estimated to cost US$67.5 billion per year [6]. Designing and implementing interventions, and nurturing environments that facilitate physical activity, are required if population health outcomes are to be optimised.

There is, however, limited published evidence on interventions that increase physical activity at the population level, with researcher-led examples mostly small scale and ineffective at maintaining any positive increases in physical activity longer term [7]. In addition, many interventions fail to engage priority populations including women and girls, physically inactive, low socio-economic and culturally and linguistically diverse, thereby reinforcing health and social inequities [8, 9]. There is consensus that a broad range of factors influence physical activity participation spanning psychological, environmental, social and policy domains [10]. Interventions that focus on all these multiple domains with a whole-systems approach are effective at reaching and engaging communities in regular physical activity [11].

The importance and urgency of reducing global levels of physical inactivity was emphasised by the WHO in its Global Action Plan on Physical Activity 2018–2030 (GAPPA). This Plan called for a 15% relative reduction in physical inactivity by 2030, supported by a whole-systems, multi-disciplinary approach. The WHO recommended that member states promote the growth of “*free, universally accessible, whole-of-community events that provide opportunities to be active in local public spaces and which aim to cultivate positive experiences and build competencies, particularly in the least active*”. parkrun was cited as an example of such an initiative.

A global charity, parkrun oversees the delivery of volunteer-led, community-based 5 k events in line with a standardised model that encourage communal physical activity, priding itself on an ethos of inclusivity [12]. parkrun events are delivered weekly, at scale across 23 countries worldwide, with 330,000 people participating in over 2200 events in areas of open space every weekend. People can take part as walkers, runners or as volunteers. Published peer reviewed literature highlights parkrun’s effectiveness in promoting physical activity, including amongst those who are less active, with a scoping review of 15 published parkrun-related studies demonstrating the organisation’s ability to engage traditionally under-represented groups [13]. Sustained improvements in fitness and physical activity levels also occurred in a dose response relationship to the frequency of parkrun attendance [13] highlighting the value of regular, sustained participation in parkrun.

Despite parkrun’s success, globally around 40% of those who register with parkrun never participate. More research is needed to better understand the demographic characteristics of people who do not participate, the reasons for non-participation and to identify and implement solutions for removing some of the barriers to facilitate participation in physical activity and help reduce health inequalities.

The objectives of this study were to i) explore the demographics of people who register for parkrun in three countries and never participate in a parkrun event, or do not continue to participate after attending once; and ii) understand the barriers to participating in parkrun amongst these people.

## Method

A cross sectional quasi-experimental study was designed. An online survey was co-developed by parkrun staff members, with input from members of the parkrun Research Board, for distribution across Australia, Ireland and the United Kingdom (UK). The three countries were chosen due to their maturity in parkrun terms, having hosted events for the longest period of time, and also each of selected countries were implementing targeted projects to promote participation. The main body of the survey was the same for all three countries, with only minor alterations to suit the country context (for example around ethnicity). It is important to acknowledge that the survey had been disseminated by parkrun to registrants in the UK and Ireland in 2015 and 2017 but 2019 was the first time the survey was distributed across the three countries; hence its primary focus here. This study was approved by the University of Sydney Human Research Ethics Committee (Reference: 2020/716).

Individuals were invited to take part in the survey if they had consented to receive communications from parkrun. Although participation in the parkrun context is defined as any individual who actively participates in the events (e.g., walks, runs or volunteers) for the purposes of this survey the definition used was narrower. The survey recipients were individuals aged 16 years and above, who had registered for parkrun and had not walked or ran, or who had completed one parkrun as a walker or runner within 2 years (2017–2019). If an individual had volunteered within 2 years but had not walked or run, they were excluded from receiving the survey. The survey was distributed by parkrun throughout September and October 2019.

Information about barriers to participation for registrants who had not walked or ran was obtained through the following question: “*We would like to know why you have not yet participated in parkrun as a walker or runner. Please tick up to three reasons that most apply to you*.” For those who had completed one parkrun as a walker or runner the question was” “*We are keen to know why you are yet to return to parkrun having successfully completed one event. Please tick up to three reasons that most apply to you.*” General open field responses were also available for voluntary completion by the survey respondent. These however were not included in the subsequent analysis.

Demographic information collected in the survey included indigenous status (Australia only) primary language spoken at home (Australia only), ethnicity (United Kingdom and Ireland) and health conditions. In Australia and the United Kingdom, for individuals who completed a survey, their postcode of residence collected at parkrun registration was matched to their survey responses. In Australia, postcode was classified into socioeconomic status (SES) quartiles of disadvantage using the Socio-Economic Indexes for Areas (SEIFA), specifically the Index of Relative Socioeconomic Disadvantage [14]. In the United Kingdom, socioeconomic status was categorised by Indices of Multiple Deprivation (IMD) [15]. Gender, age, and activity status, collected at parkrun registration, were matched to the individual survey responses. An active registrant is defined as achieving 30 min or more on 4 or more days of the week and an inactive registrant 0 days achieving 30 min or more.

### Data analysis

As the survey sample across Australia, Ireland and the United Kingdom differed significantly from the distribution for age and gender from all parkrun registrants who did not participate or only participated in one event, sample weights were calculated using iterative proportional fitting. These weights were applied to all subsequent analyses. Descriptive statistics, including raw frequencies and weighted proportions were calculated for those who did not participate in any parkrun events and those who participated in only one event. Logistic regression models were conducted to determine which demographic characteristics were associated with each barrier, adjusting for age, sex, socioeconomic status, and physical activity at registration. All analyses were conducted in SAS Enterprise Guide 9.4 (SAS Institute, Cary, NC, USA).

## Results

During 2017–2019 there were 680,255 parkrun registrants. Of these, 293,542 (43%) did not participate in a parkrun event (Table 1) and 147,148 (22%) participated in only one parkrun event (Table 2).

**Table 1:** Demographic characteristics of those who registered for parkrun and did not participate using registration data and survey data across three countries

	Registered for parkrun		Registered for parkrun and did not participate in an event		Registered for parkrun and did not participate in an event and completed a survey	
	N	%	N	%	N	% ^a^
All people	680,255	100	293,542	43.2	3094	1.1
Age						
16–34	297,384	43.7	138,863	47.3	522	44.7
35–44	175,460	25.8	78,004	26.6	874	28.0
45–54	132,053	19.4	51,094	17.4	950	18.8
55–64	57,606	8.5	19,558	6.7	553	6.7
65 and Over	17,752	2.6	6023	2.1	193	1.9
Gender						
Male	301,627	44.3	123,728	42.2	1045	42.3
Female	378,628	55.7	169,714	57.8	2048	57.7
Physical Activity level^b^						
Less than once per week	70,892	10.6	36,735	12.8	973	32.1
Once per week	101,988	15.3	46,402	16.1	252	7.6
Twice per week	157,154	23.5	65,987	23.0	394	11.6
Three times per week	191,543	28.7	78,135	27.2	528	17.4
More than four times per week	146,932	22.0	60,101	20.9	946	31.3

^a^Proportions are weighted

^b^an active registrant is defined as achieving 30 min or more on 4 or more days of the week and an inactive registrant 0 days achieving 30 min or more

**Table 2:** Demographic characteristics of those who registered for parkrun and participated in one event using registration data and survey data across three countries

	Registered for parkrun		Registered for parkrun and only participated in one event		Registered for parkrun and only participated in one event and completed a survey	
	N	%	N	%	N	% ^a^
All	680,255	100	147,148	21.6	2673	1.8
Age						
16–34	297,384	43.7	67,842	46.1	515	49.3
35–44	175,460	25.8	36,624	24.9	737	25.9
45–54	132,053	19.4	26,868	18.3	760	16.4
55–64	57,606	8.5	12,078	8.2	469	6.3
65 and Over	17,752	2.6	3736	2.5	192	2.1
Gender						
Male	301,627	44.3	64,612	43.9	874	41.4
Female	378,628	55.7	82,636	56.2	1799	58.6
Physical Activity level						
Less than once per week	70,892	10.6	13,210	9.1	467	18.6
Once per week	101,988	15.3	20,919	14.4	225	8.3
Twice per week	157,154	23.5	34,768	24.0	374	14.0
Three times per week	191,543	28.7	42,738	29.5	592	20.6
Four or more times per week	146,932	22.0	33,248	22.9	1015	38.5

^a^Proportions are weighted

The 16–34-year age group had the highest number of registrants (43.7%) and also the highest proportion of those who registered but did not participate (47.3%) or registered and only participated in one event (46.1%). Compared to males, a higher proportion of females registered for parkrun (55.7%) but a higher proportion of females than males did not participate at all (57.8%) or only participated in one event (56.2%). Compared to the total proportion of inactive people who registered for parkrun (10.6%), a higher proportion of those who registered but did not participate were inactive compared to those who did one or more 30 min bout of exercise per week when they registered for parkrun (12.8%).

The survey sample consisted of 3094 registrants who had not participated in a parkrun event and 2673 who had participated in one parkrun event, but not returned. The weighted proportions of the survey sample are like the population from which they were drawn. For example, of those who registered and did not participate, 57.8% were female and females comprised 57.7% of the survey sample.

### Barriers

The main barriers for those who did not participate in an event were the start time being inconvenient (20%), feeling too unfit (13%), injury/illness (12%), no time (12%) and childcare obligations (10%). The main barriers for those who had participated in one event were the start time being inconvenient (24%), no time (21%), injury/illness (15%) and childcare obligations (14%). These barriers are presented in more detail below and in Tables 3 and 4.

**Table 3:** Adjusted odds of reporting barriers across demographic characteristics for those who did not participate in any parkrun events

	Start time inconvenient (20%)	Feels too unfit (13%)	Injury/illness (12%)	No time (12%)	Childcare obligations (10%)	Not sure what to expect (9%)	Don’t want to go by myself (9%)	Forgot barcode (7%)	Too far (7%)	Concerned about running in public (5%)
	OR (95% CIs)	OR (95% CIs)	OR (95% CIs)	OR (95% CIs)	OR (95% CIs)	OR (95% CIs)	OR (95% CIs)	OR (95% CIs)	OR (95% CIs)	OR (95% CIs)
Age category										
16–24	Reference	Reference	Reference	Reference	Reference	Reference	Reference	Reference	Reference	Reference
25–34	1.71 (0.97, 3.02)	1.15 (0.61, 2.19)	0.66 (0.34, 1.28)	0.67 (0.38, 1.2)		1.34 (0.66, 2.72)	0.98 (0.51, 1.86)	0.73 (0.37, 1.44)	1.9 (0.77, 4.69)	2 (0.74, 5.38)
35–44	1.76 (1.03, 3.02) *	0.92 (0.5, 1.68)	0.71 (0.39, 1.3)	0.62 (0.36, 1.05)		1.06 (0.54, 2.08)	0.51 (0.28, 0.95) *	0.48 (0.25, 0.91) *	1.52 (0.64, 3.63)	0.77 (0.29, 2.06)
45–54	1.26 (0.74, 2.17)	1.14 (0.63, 2.07)	1.34 (0.75, 2.4)	0.43 (0.25, 0.73) **		0.87 (0.44, 1.71)	0.51 (0.28, 0.95) *	0.4 (0.21, 0.76) **	1.03 (0.42, 2.48)	1.24 (0.47, 3.23)
55–64	1.04 (0.59, 1.83)	0.82 (0.44, 1.54)	1.83 (1.01, 3.32) *	0.33 (0.18, 0.6) **		0.97 (0.48, 1.96)	0.48 (0.25, 0.93) *	0.25 (0.12, 0.52) ***	1.31 (0.53, 3.23)	0.94 (0.34, 2.59)
65+	0.97 (0.51, 1.85)	1 (0.49, 2.02)	2.22 (1.16, 4.23) *	0.33 (0.16, 0.68) **		0.47 (0.18, 1.18)	0.63 (0.29, 1.37)	0.32 (0.13, 0.79) *	1.18 (0.42, 3.32)	0.49 (0.13, 1.88)
Gender										
Male	Reference	Reference	Reference	Reference	Reference	Reference	Reference	Reference	Reference	Reference
Female	0.95 (0.78, 1.16)	1.47 (1.15, 1.89) **	0.73 (0.59, 0.91) **	0.74 (0.58, 0.96) *	1.2 (0.92, 1.58)	1.72 (1.28, 2.33) ***	2.65 (1.86, 3.77)	0.78 (0.56, 1.09)	1.2 (0.87, 1.67)	2.82 (1.73, 4.58)
Socioeconomic status quartile										
1st	1.12 (0.81, 1.55)	1.02 (0.7, 1.47)	1.01 (0.7, 1.47)	1.29 (0.87, 1.93)	0.66 (0.42, 1.04)	0.84 (0.52, 1.35)	1.26 (0.81, 1.98)	1.13 (0.65, 1.97)	1.41 (0.84, 2.34)	0.94 (0.51, 1.72)
2nd	1.03 (0.77, 1.36)	1.08 (0.79, 1.48)	1.14 (0.84, 1.55)	0.72 (0.49, 1.08)	0.83 (0.57, 1.21)	1.20 (0.83, 1.74)	1.26 (0.86, 1.86)	1.47 (0.94, 2.31)	1.58 (1.02, 2.43) *	1.14 (0.69, 1.88)
3rd	1.22 (0.96, 1.57)	0.86 (0.64, 1.15)	1.09 (0.83, 1.44)	1.35 (0.99, 1.85)	0.97 (0.7, 1.35)	1.14 (0.81, 1.61)	1.16 (0.8, 1.67)	1.11 (0.71, 1.72)	1.32 (0.87, 1.98)	0.97 (0.6, 1.56)
4th	Reference	Reference	Reference	Reference	Reference	Reference	Reference	Reference	Reference	Reference
Number of days of physical activity per week										
0	0.69 (0.50, 0.95) *	3.9 (2.75, 5.53)	2.02 (1.45, 2.8)	0.78 (0.5, 1.21)	1.13 (0.74, 1.72)	0.99 (0.62, 1.56)	1.63 (1.05, 2.53) *	0.71 (0.39, 1.29)	0.86 (0.51, 1.44)	2.58 (1.48, 4.51) ***
1	0.87 (0.63, 1.20)	3.9 (2.73, 5.58)	1.21 (0.83, 1.76)	0.94 (0.61, 1.45)	1.38 (0.91, 2.11)	1.17 (0.74, 1.83)	1.48 (0.94, 2.35)	0.96 (0.55, 1.68)	0.65 (0.36, 1.17)	1.56 (0.82, 2.98)
2	0.76 (0.58, 1.01)	2.97 (2.14, 4.12)	1.51 (1.12, 2.05) **	1.06 (0.74, 1.52)	1.22 (0.84, 1.78)	1.83 (1.29, 2.59) ***	1.57 (1.06, 2.32) *	0.71 (0.42, 1.19)	0.9 (0.58, 1.41)	2.55 (1.54, 4.22) ***
3	0.71 (0.55, 0.92) **	1.95 (1.41, 2.69)	1.25 (0.94, 1.68)	1.06 (0.77, 1.47)	1.31 (0.94, 1.82)	1.23 (0.87, 1.74)	1.46 (1.01, 2.10) *	1.17 (0.79, 1.74)	1.15 (0.79, 1.68)	1.81 (1.10, 2.98) *
4+	Reference	Reference	Reference	Reference	Reference	Reference	Reference	Reference	Reference	Reference

Socioeconomic status was only possible to determine for participants in the United Kingdom and Australia.

*p < 0.05, **p < 0.01, ***p < 0.001

**Table 4:** Adjusted odds of reporting barriers across demographic characteristics for those who participated in one parkrun event

	Start time Inconvenient (24%)	Feels Too unfit (4%)	Injury/illness (15%)	No time (21%)	Childcare obligations (14%)	Don’t want to go by myself (8%)	Forgot barcode (2%)	Too far (9%)	Concerned about running in public (3%)
Age category									
16–24	Reference	Reference	Reference	Reference	Reference	Reference	Reference	Reference	Reference
25–34	1.01 (0.64, 1.57)	0.85 (0.36, 2.00)	3.48 (1.45, 8.35) **	0.68 (0.44, 1.06)	20.61 (2.81, 151.25) ***	1.01 (0.53, 1.9)	1.2 (0.56, 2.55)	0.74 (0.40, 1.38)	0.92 (0.35, 2.41)
35–44	0.76 (0.50, 1.15)	0.38 (0.16, 0.90) *	4.37 (1.88, 10.17) ***	0.44 (0.29, 0.67) ***	56.64 (7.86, 408.13) ***	0.39 (0.2, 0.74) **	1.01 (0.49, 2.05)	0.54 (0.30, 0.97) *	0.44 (0.17, 1.15)
45–54	0.64 (0.42, 0.98) *	0.64 (0.28, 1.43)	5.37 (2.32, 12.44) ***	0.43 (0.29, 0.66) ***	19.05 (2.63, 137.94) ***	0.48 (0.26, 0.9) *	0.52 (0.24, 1.10)	0.66 (0.37, 1.17)	0.36 (0.13, 0.97) *
55–64	0.48 (0.31, 0.76) **	0.87 (0.38, 2.00)	8.69 (3.73, 20.24) ***	0.29 (0.18, 0.46) ***	2.32 (0.29, 18.71)	0.47 (0.24, 0.93) *	0.62 (0.28, 1.35)	0.46 (0.24, 0.86) *	0.55 (0.2, 1.52)
65+	0.42 (0.24, 0.74) **	0.44 (0.14, 1.39)	9.96 (4.14, 24.00) ***	0.22 (0.12, 0.41) ***	2.88 (0.32, 26.08)	0.84 (0.40, 1.79)	0.43 (0.15, 1.21)	0.66 (0.32, 1.36)	
Gender									
Male	Reference	Reference	Reference	Reference	Reference	Reference	Reference	Reference	Reference
Female	0.83 (0.68, 1.02)	2.41 (1.43, 4.04) ***	0.84 (0.68, 1.03)	0.53 (0.43, 0.66) ***	0.97 (0.75, 1.26)	2.03 (1.37, 3.00) ***	0.70 (0.49, 1.00) *	1.12 (0.82, 1.53)	4.26 (1.82, 9.97) ***
Socioeconomic status quartile									
1st	0.99 (0.71, 1.38)	2.08 (1.15, 3.76) *	1.43 (1.03, 1.99) *	0.78 (0.53, 1.17)	0.49 (0.31, 0.78) ***	1.49 (0.92, 2.42)	1.06 (0.60, 1.89)	1.04 (0.63, 1.71)	2.42 (1.09, 5.35) *
2nd	0.87 (0.66, 1.14)	1.2 (0.68, 2.11)	1.17 (0.89, 1.54)	1.33 (1.00, 1.78)	0.71 (0.50, 1.01)	1.14 (0.75, 1.73)	1.04 (0.65, 1.67)	1.11 (0.75, 1.64)	1.39 (0.64, 3.00)
3rd	0.89 (0.69, 1.15)	1.13 (0.66, 1.94)	1.04 (0.81, 1.35)	1.07 (0.81, 1.41)	1.00 (0.74, 1.34)	0.74 (0.48, 1.15)	0.72 (0.45, 1.17)	0.86 (0.59, 1.26)	1.59 (0.78, 3.25)
4th	Reference	Reference	Reference	Reference	Reference	Reference	Reference	Reference	Reference
Number of days of physical activity per week									
0	0.63 (0.42, 0.93) *	4.28 (2.41, 7.59)	2.91 (2.11, 4.02) ***	1.55 (1.06, 2.25) *	1.08 (0.69, 1.68)	1.05 (0.57, 1.94)	0.30 (0.11, 0.83) *	0.72 (0.4, 1.29)	2.53 (1.14, 5.63) *
1	1.09 (0.78, 1.52)	2.23 (1.14, 4.33) *	1.56 (1.1, 2.23) *	1.59 (1.10, 2.3) *	1.11 (0.72, 1.71)	1.68 (1.00, 2.82)	0.43 (0.18, 1.02)	1.02 (0.61, 1.7)	1.50 (0.61, 3.66)
2	0.82 (0.61, 1.10)	1.95 (1.09, 3.50) *	1.32 (0.97, 1.78)	1.38 (1.01, 1.89) *	1.11 (0.78, 1.58)	1.71 (1.11, 2.63) *	1.00 (0.60, 1.66)	0.95 (0.62, 1.46)	2.19 (1.10, 4.33) *
3	0.94 (0.74, 1.20)	1.58 (0.91, 2.73)	1.32 (1.02, 1.71) *	1.14 (0.86, 1.51)	1.23 (0.91, 1.67)	1.27 (0.84, 1.90)	1.14 (0.75, 1.75)	1.12 (0.79, 1.59)	1.22 (0.60, 2.50)
4	Reference	Reference	Reference	Reference	Reference	Reference	Reference	Reference	Reference

Socioeconomic status was only possible to determine for participants in the United Kingdom and Australia.

*p < 0.05, **p < 0.01, ***p < 0.001

#### Start time being inconvenient

The start time being inconvenient was reported as a barrier by 20% of those who registered but did not participate in an event and 24% of those who registered and only participated in one event. For those who did not participate, compared with 16–24-year-olds, 35–44-year-olds were more likely to report the start time being inconvenient (OR: 1.76, 95% CIs 1.03, 3.02). For those who participated once, compared with 16–24-year-olds, people aged over 45 were less likely to report the start time being inconvenient (45–54 years OR: 0.64, 95% CIs 0.42, 0.98; 55–64 years OR: 0.48, 95% CIs 0.31, 0.76; 65+ years OR: 0.42, 95% CIs 0.24, 0.74). Compared with active registrants, registrants who were inactive (0 days) were less likely to report that the start time was a barrier (no events OR: 0.69, 95% CIs 0.50, 0.95; one event OR: 0.63, 0.42, 0.93).

#### Feeling too unfit or feeling unable to complete the 5 k

Feeling too unfit was reported as a barrier by 13% of those who registered but did not participate in an event and 4% of those who registered and only participated in one event. Compared with males, females were more likely to report feeling too unfit as a barrier (no events OR: 1.47, 95% CIs 1.15, 1.89; one event OR: 2.41, 95% CIs 1.43, 4.04). Compared with an active registrant, inactive registrants were more likely to report feeling too unfit (no events 1 day PA OR: 3.90, 95% CIs 2.75, 5.53; one event 0 days PA OR: 4.28, 95% CIs 2.41, 7.59). For those who participated once, compared with registrants who live in the least disadvantaged area, those who live in the most disadvantaged area were more likely to report feeling too unfit (OR: 2.08, 95% CIs 1.15, 3.76).

#### Pain, injury or illness or other health reasons

Pain, injury, or illness was reported as a barrier for 12% of those who registered but did not participate in an event and 15% of those who registered and only participated in one event. The older age groups were most likely to report pain, injury, or illness (no events 65+ years OR: 2.22, 95% CIs 1.16, 4.23; one event 65+ years OR: 9.96, 95% CIs 4.14, 24.00). For those who did not participate, females were less likely than males to report pain, injury, or illness (OR: 0.73, 95% CIs 0.59, 0.91). Compared to active registrants, those who were not active were more likely to report pain, injury, or illness (no events OR: 2.02, 95% CIs 1.45, 2.80; one event OR: 2.91, 95% CIs 2.11, 4.02). For those who participated once, registrants living in the most disadvantaged area were more likely to report pain, injury or illness compared to those living in the least disadvantaged area (OR: 1.43, 95% CIs 1.03, 1.99).

#### Not having time

Not having time was reported as a barrier for 12% of those who registered but did not participate in an event and 21% of those who registered and only participated in one event. Older registrants were the least likely to report time as a barrier (no events 65+ years OR: 0.33, 95% CIs 0.16, 0.68; one event 65+ years OR: 0.22, 95% CIs 0.12, 0.41). Compared with males, females were less likely to report not having time (no events OR: 0.74, 95% CIs 0.58, 0.96; one event OR: 0.53, 95% CIs 0.43, 0.66). For those who participated once, compared with active registrants, those who were inactive, or active 1 or 2 days were more likely to report time as a barrier (inactive OR: 1.55, 95% CIs 1.06, 2.25; 1 day PA OR: 1.59, 95% CIs 1.10, 2.30; 2 days PA OR: 1.38, 95% CIs 1.01, 1.89).

#### Childcare obligations

Childcare obligations were reported as a barrier for 10% of those who registered but did not participate in an event and 14% of those who registered and only participated in one event. For those who participated in one event, registrants living in the most disadvantaged areas were less likely to report childcare obligations compared with registrants living in the least disadvantaged areas (OR: 0.49, 95% CIs 0.31, 0.78).

## Discussion

Increasing population physical activity levels is a global priority. Physical activity interventions are most effective when they impact underlying mechanisms that influence physical activity behaviours. Physical activity research to date has focused on the determinants for physical activity to inform population policies and programs. Understanding the barriers to physical activity participation are essential in enabling interventions, like parkrun, to maximise reach and public health impact of delivery.

parkrun events are a successful, regular, community-based, and community-led physical activity intervention delivered globally at scale [12]. This is the first published research study with the dual objectives of; i) Understanding the demographic characteristics of people who register for parkrun and do not participate, or do not sustain participation in parkrun, across three well-established parkrun countries; ii) Understanding the self-reported barriers to participating in parkrun across three parkrun territories; Australia, Ireland, and the United Kingdom. This study helps to fill the research gap identified by Grunseit et al., [13] with findings being valuable and applicable to the global parkrun community, policymakers, practitioners, and academics who all seek effective and scalable ways to support more people to be physically active to improve population health and wellbeing.

Often age and gender emerge from physical activity evidence as consistent demographic correlates of physical activity behaviour. Physical activity participation is consistently higher in men than women, and often inversely related with age [4, 15]. Interestingly most people who registered for parkrun across Australia, Ireland and the United Kingdom between 2017 and 2019 were aged 16–34 years old and were female, reinforcing the good reach at population level and parkrun’s ability to challenge participation inequalities [13].

Between 2017 and 19, 43% of individuals who registered for parkrun across Australia, Ireland and the United Kingdom did not participate in a parkrun event, with 22% of those only participating in one event. These people, along with survey respondents, were mostly female, aged between 16 and 34 years and were physically inactive at the point of parkrun registration. The representativeness of this study provides confidence that the data collected through the surveys which can be extrapolated transferring learning across the parkrun community and beyond.

Individuals who were physically inactive at the point of registration, and younger registrants (particularly under aged 35 years) were least likely to convert to participating more than once. While the younger groups and females are more likely to not participate and not to return, our data suggests that for the least active the greatest issue is attending parkrun for the first time. Amongst this group, once they have completed one parkrun, they are not significantly less likely to return. Younger registrants across three countries are also less likely to sustain participation in parkrun. This suggests that additional support immediately following registration could help encourage people to attend parkrun for the first time. Targeted communication tailored for different ages could also engage the younger demographic. Promoting the volunteer roles also available at parkrun could be another help bridge the gap between registering and actively participating.

The most frequently cited self-reported barriers for attending parkrun were similar across people who never attended a parkrun event and people who only attended once. Self-reported barriers included: perceptions of feeling too unfit to participate in 5 km, pain, injury or illness or other health reasons, not having time, childcare obligations, with the most frequently cited barrier being that the start time was inconvenient.

Arguably a successful component of parkrun’s global scalability has been the consistent mode of delivery; always free, weekly and on Saturday mornings. This suggests that parkrun could consider placing emphasis on the opportunity to take part as a family, highlight the inclusive culture of parkrun and reinforce the breadth of health wellbeing and social benefits associated with engagement to counter the perceived challenges associated with participation [13]. There must also be recognition that due to the consistent start time for parkrun, irrespective of what parkrun does, this time just might not suit some people.

The range of barriers reported here align with existing evidence that recognises a broad range of factors influencing physical activity behaviours, often spanning across psychological, environmental, social, and policy domains [10]. Individual psychological factors such as confidence and perceived competence are shown to clearly predict and affect physical activity participation [16, 17] whilst social environmental such as emotional and logistical support, expressed here through childcare commitments, also play a role [18].

The clearest correlates and determinants of adult physical activity behaviour include health status and self-efficacy [19]. Within this parkrun data, pain, injury and ill health was a barrier to participating in parkrun, associated with increasing age. Whilst research has shown regular participation in parkrun has a positive impact on physical and mental health in the short and long term [13, 20, 21], further work with individuals experiencing pain or with chronic health conditions is needed to help understand how this barrier can be alleviated for this population group. Alternatively exploring partnerships with stakeholders such as health professionals could support people affected by pain and chronic illness to become physically active through parkrun. In the United Kingdom and Ireland, the parkrun practice initiative was launched in 2018 to link parkrun events with primary care to raise awareness of parkrun and increase participation in local parkrun events by staff and patients [22].

The perception of feeling too unfit to participate in parkrun or complete the 5 km was the most cited barrier amongst females in this study. This speaks to a psychological barrier primarily experienced by females, most likely underpinned by low self-efficacy to be physically active [23]. Low levels of self-efficacy have been repeatedly shown throughout literature to be associated with lower levels of physical activity [23, 24]. Often strategies to increase self-efficacy have focused on the integration of evidence-based behaviour change techniques within program delivery such as positive feedback and positive inclusive environments. The use of public education programs specifically aimed at overcoming the psychological barriers for women to be active e.g., Sport England’s ‘*This Girl Can’* campaign, have received government investment, yet there is no published evidence of impact. parkrun could explore further work with women experiencing psychological barriers to co-design the best approach for enabling their increased future engagement.

### Strengths and limitations

A significant strength of this research is that it is one of the first published global studies, across three countries well-established in delivering parkrun events, to understand the demographic characteristics of people who register for parkrun and do not start or sustain participation in parkrun and the barriers to participation reported by these people report. Further, the initial large sample was weighted to the population of all parkrun registrants who did not participate or only participated in one event to provide a representative sample. However, there are limitations. It is important to remember that registration at parkrun requires technological access and skills, which could create a bias regarding population reach. This study was cross-sectional in nature, meaning causal inferences cannot be made. Socio-economic status was only available for the survey data in Australia and United Kingdom. The wording and content of the surveys distributed to people who never attended and people who only attended parkrun once was different and open field text responses were not qualitatively analysed. Whilst, further longitudinal and qualitative research is needed. These findings do however provide valuable information to parkrun, and other policymakers, practitioners, and academics, focused on increasing participation in community based physical activity opportunities.

## Conclusions and future research

This study acknowledges the population reach of parkrun across Australia, Ireland and the United Kingdom but also reinforces inequalities in participation. Individuals aged 16–34, females and those who were physically inactive were more likely to not participate or not return to parkrun having completed one event. Bridging the gap between parkrun registration and attending once was a significant issue amongst younger females and physically inactive at the point of registration with parkrun. Overall, inconvenient start time was the most frequently reported barrier to participating amongst parkrun registrants, with women more likely than men to report the psychological barrier of feeling too unfit to participate. Co-creating strategies with and for people living with a chronic disease, women, and young adults physically inactive people, could increase physical activity participation within parkrun. Further in-depth qualitative work that elicits rich community experiences could also be beneficial.

## Supplementary Information

**Additional file 1.**

## Data Availability

The datasets used and/or analysed during the current study are available from the corresponding author on reasonable request.
