# Supplementary material for: Barriers to initiating and maintaining participation in parkrun
Source: BMC Public Health. 2022 Jan 13;22:83. doi: 10.1186/s12889-022-12546-w (PMC8759213; doi:10.1186/s12889-022-12546-w)
Supplement: Supplementary file 1 — Additional file 1. [file 12889_2022_12546_MOESM1_ESM.docx]

**Supplemental Table 1** Demographic characteristics of those who registered for parkrun and participated in one event and did not participate using registration data across three countries

|  | All registrants | | | | | | | | No events | | | | | | | | One event | | | | | | | |
| --- | --- | --- | --- | --- | --- | --- | --- | --- | --- | --- | --- | --- | --- | --- | --- | --- | --- | --- | --- | --- | --- | --- | --- | --- |
|  | United Kingdom | | Australia | | Ireland | | Worldwide | | United Kingdom | | Australia | | Ireland | | Worldwide | | United Kingdom | | Australia | | Ireland | | Worldwide | |
|  | N | % | N | % | N | % | N | % | N | % | N | % | N | % | N | % | N | % | N | % | N | % | N | % |
| All | 508,212 | 75 | 131,800 | 19 | 40,243 | 6 | 680,255 | 100 | 222,065 | 76 | 52,404 | 18 | 19,073 | 6 | 293,542 | 100 | 110,094 | 75 | 28,508 | 19 | 8,546 | 6 | 147,148 | 100 |
| Age |  |  |  |  |  |  |  |  |  |  |  |  |  |  |  |  |  |  |  |  |  |  |  |  |
| 16-24 | 75,255 | 15 | 19,466 | 15 | 4,513 | 11 | 99,234 | 15 | 34,616 | 16 | 8,320 | 16 | 2,183 | 11 | 45,119 | 15 | 18,604 | 17 | 5,101 | 18 | 1,174 | 14 | 24,879 | 17 |
| 25-34 | 150,656 | 30 | 35,945 | 27 | 11,549 | 29 | 198,150 | 29 | 71,756 | 32 | 16,016 | 31 | 5,972 | 31 | 93,744 | 32 | 32,711 | 30 | 7,765 | 27 | 2,487 | 29 | 42,963 | 29 |
| 35-44 | 126,750 | 25 | 36,135 | 27 | 12,575 | 31 | 175,460 | 26 | 57,532 | 26 | 14,304 | 27 | 6,168 | 32 | 78,004 | 27 | 26,581 | 24 | 7,487 | 26 | 2,556 | 30 | 36,624 | 25 |
| 45-54 | 99,656 | 20 | 24,678 | 19 | 7,719 | 19 | 132,053 | 19 | 38,871 | 18 | 8,899 | 17 | 3,324 | 17 | 51,094 | 17 | 20,346 | 18 | 4,962 | 17 | 1,560 | 18 | 26,868 | 18 |
| 55-64 | 43,719 | 9 | 10,884 | 8 | 3,003 | 7 | 57,606 | 8 | 14,985 | 7 | 3,461 | 7 | 1,112 | 6 | 19,558 | 7 | 9,254 | 8 | 2,251 | 8 | 573 | 7 | 12,078 | 8 |
| 65 and Over | 12,176 | 2 | 4,692 | 4 | 884 | 2 | 17,752 | 3 | 4,305 | 2 | 1,404 | 3 | 314 | 2 | 6,023 | 2 | 2,598 | 2 | 942 | 3 | 196 | 2 | 3,736 | 3 |
| Gender |  |  |  |  |  |  |  |  |  |  |  |  |  |  |  |  |  |  |  |  |  |  |  |  |
| Male | 229,901 | 45 | 54,635 | 41 | 17,091 | 42 | 301,627 | 44 | 96,123 | 43 | 20,037 | 38 | 7,568 | 40 | 123,728 | 42 | 48,660 | 44 | 12,297 | 43 | 3,655 | 42 | 64,612 | 44 |
| Female | 278,311 | 55 | 77,165 | 59 | 23,152 | 58 | 378,628 | 56 | 125,942 | 57 | 32,367 | 62 | 11,405 | 60 | 169,714 | 58 | 61,434 | 56 | 16,211 | 57 | 4,991 | 58 | 82,636 | 56 |
| Physical Activity level |  |  |  |  |  |  |  |  |  |  |  |  |  |  |  |  |  |  |  |  |  |  |  |  |
| Less than once per week | 53,283 | 11 | 14,034 | 11 | 3,575 | 9 | 70,892 | 11 | 28,296 | 13 | 6,450 | 13 | 1,989 | 11 | 36,735 | 13 | 9,741 | 9 | 2,807 | 10 | 662 | 8 | 13,210 | 9 |
| Once per week | 77,727 | 16 | 18,464 | 14 | 5,797 | 15 | 101,988 | 15 | 35,884 | 16 | 7,677 | 15 | 2,841 | 15 | 46,402 | 16 | 16,032 | 15 | 3,746 | 13 | 1,141 | 13 | 20,919 | 14 |
| Twice per week | 120,888 | 24 | 26,421 | 20 | 9,845 | 25 | 157,154 | 24 | 51,193 | 24 | 10,317 | 20 | 4,477 | 24 | 65,987 | 23 | 26,868 | 25 | 5,681 | 20 | 2,219 | 26 | 34,768 | 24 |
| Three times per week | 145,544 | 29 | 34,287 | 27 | 11,712 | 30 | 191,543 | 29 | 59,465 | 27 | 13,301 | 26 | 5,369 | 29 | 78,135 | 27 | 32,670 | 30 | 7,491 | 27 | 2,577 | 30 | 42,738 | 29 |
| Four or more times per week | 102,365 | 20 | 36,074 | 28 | 8,493 | 22 | 146,932 | 22 | 42,779 | 20 | 13,482 | 26 | 3,840 | 21 | 60,101 | 21 | 23,115 | 21 | 8,238 | 29 | 1,895 | 22 | 33,248 | 23 |
